# Comparative Efficacy of CK2 Inhibitors CX-4945 and SGC-CK2-2 on CK2 Signaling

**DOI:** 10.3390/ijms262010006

**Published:** 2025-10-14

**Authors:** Francesca Noventa, Rina Venerando, Valentina Bosello Travain, Mauro Salvi

**Affiliations:** 1 Department of Biomedical Sciences, University of Padova, Via U. Bassi 58/B, 35131 Padova, Italy; 2 Department of Molecular Medicine, University of Padova, Via Gabelli 63, 35121 Padova, Italy

**Keywords:** protein phosphorylation, kinase inhibitors, CX-4945, SGC-CK2-2

## Abstract

The pleiotropic kinase CK2 plays a crucial role in numerous cellular processes and is frequently deregulated in human diseases. Specifically, elevated CK2 expression and/or activity have been observed in human cancers, thus rendering its inhibition a promising pharmacological strategy for treating malignancies. The most widely used CK2 inhibitor, CX-4945 (Silmitarsetib), was developed by Cylene Pharmaceuticals in 2010. It has been tested in clinical trials for various cancers and, more recently, as a potential therapy for COVID-19 patients. However, it has been demonstrated that CX-4945’s specificity is limited, as CX-4945 also inhibits other kinases beyond CK2. A recently developed derivative of CX-4945, SGC-CK2-2, has demonstrated enhanced specificity compared with CX-4945, albeit with reduced potency. In this study, we conducted a detailed analysis of the effects of SGC-CK2-2 in two cancer cell lines, comparing its efficacy with CX-4945 in inhibiting CK2 signaling and in cell death induction. The findings of this study demonstrate the differential sensitivity of CK2 phospho-substrates to these inhibitors, thus indicating that complete inhibition of a single phosphosite, such as S129 Akt, is insufficient to fully suppress CK2 signaling. Furthermore, the results suggest that partial CK2 inhibition with the suppression of the most sensitive phosphosites does not significantly impact cell viability, while a near-complete suppression of CK2 signaling affects cell viability and leads to cell death induction.

## 1. Introduction

The wide involvement of CK2 in a multitude of cellular processes and its deregulated expression in various human pathologies [1,2,3,4,5,6,7,8] have driven significant efforts to develop modulators of its activity, with particular emphasis on the design of small chemical inhibitors. The use of CK2 inhibitors has been extensively studied in cancer, since CK2 is considered a promising target for treating various human malignancies. Indeed, CK2 expression and activity are upregulated in several types of tumors, contributing to cancer pathogenesis through multiple mechanisms [1,2,3,9,10,11,12]. Detailed information about the development of CK2 inhibitors is available in two recent reviews [13,14].

The most widely used CK2 inhibitor, CX-4945 (Silmitarsetib), was developed by Cylene Pharmaceuticals in 2010 [15] and granted designation as an orphan drug by the U.S. Food and Drug Administration [16]. CX-4945 demonstrated an excellent pharmacokinetic profile [17] and proved highly suitable for in vivo applications [13]. CX-4945 entered in different clinical trials, in particular for treating human tumors [18] and as a potential therapeutic option for patients with severe acute respiratory syndrome (SARS-CoV-2) [5]. It remains the inhibitor of choice for in vivo treatments, either as a standalone therapy or in combination with other approaches [18]. Despite its extensive use, CX-4945 has been shown to exhibit several off-target effects, as it also inhibits other kinases at concentrations close to those required for CK2 inhibition [19,20,21,22]. In particular, in a recent phosphoproteomic analysis comparing the effects of CX-4945 and a more specific CK2 inhibitor (see below), the phosphosites that decreased following CX-4945 treatment were enriched in the S/T–E/D–x–E/D motif, which is characteristic of CK2 substrates [23]. However, they also showed an enrichment of proline at the +1 position, a hallmark of substrates phosphorylated by proline-directed kinases [20]. Notably, the presence of proline at the +1 position is detrimental to CK2 phosphorylation [24], suggesting that CX-4945 also affects members of this kinase family and likely extends to multiple proline-directed kinases. The search for more-specific inhibitors has led to the development by Axtman’s group of SGC-CK2-1, the most potent and specific CK2 inhibitor to date [25]. Broad kinase profiling confirmed that SGC-CK2-1 exhibits remarkable selectivity, targeting only CK2α and CK2α’, while maintaining an approximately 100-fold selectivity window over the next most inhibited kinase, DYRK2 [25]. The previously mentioned comparative phosphoproteomic analysis of cells treated with SGC-CK2-1 and CX-4945 provided strong evidence for the superior selectivity of SGC-CK2-1 in cells. Specifically, only 15% and 5% of the phosphopeptides significantly downregulated after 4 h and 24 h of CX-4945 treatment, respectively, were determined to be CSNK2A1 dependent, whereas as many as 55% of the downregulated phosphopeptides at both 4 h and 24 h following SGC-CK2-1 treatment were identified as CSNK2A1 dependent [20]. However, SGC-CK2-1’s structural properties prevent its application in in vivo studies [13]. Efforts to optimize SGC-CK2-1 for in vivo applications were limited by its moderate aqueous solubility and rapid metabolic clearance, as 60% of the compound was degraded in vitro in mouse liver microsomes after just 30 min of incubation [26]. More recently, the same research group aimed to improve the kinase selectivity of CX-4945, resulting in the development of SGC-CK2-2, a naphthyridine-based CK2 chemical probe [27]. SGC-CK2-2 has demonstrated exceptional selectivity, inhibiting only CK2α and CK2α’, with an approximately 200-fold selectivity margin over the next most strongly inhibited kinase, HIPK2 [27], despite a reduced potency in CK2 inhibition in cells compared with SGC-CK2-1 (CK2α NanoBRET IC50: 36 nM for SGC-CK2-1 vs. 920 nM for SGC-CK2-2). Importantly, SGC-CK2-2 exhibits markedly improved aqueous kinetic solubility relative to SGC-CK2-1 [13]. However, to date, SGC-CK2-2 has not yet been evaluated in vivo, and its pharmacokinetic properties, bioavailability, and potential efficacy in animal models remain to be investigated. Preliminary proliferation assays suggested that SGC-CK2-2 lacks antiproliferative effects [27], raising the possibility that the cell death attributed to CK2 inhibition by CX-4945 may instead stem from its off-target effects [27]. This idea, previously introduced during the development of SGC-CK2-1 [25], has been a topic of considerable debate [28]. In this study, we performed a comparative analysis of the new CX-4945 derivative SGC-CK2-2 against the parental compound. We compared the effects of the two inhibitors on CK2 signaling as well as their impact on cell proliferation and cell death.

## 2. Results

**Figure 1:**
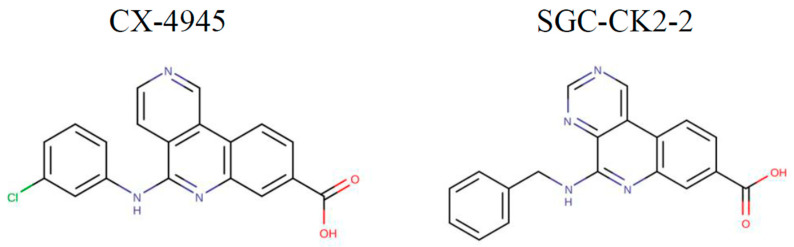
Chemical structure of CX-4945 and SGC-CK2-2. The differences between the two molecules are shown in color. NanoBRET data and selectivity data are available in [27].

In this study, the effect of the well-known CK2 inhibitor, CX-4945, was compared with that of SGC-CK2-2, a modified version of the molecule that exhibited improved kinase selectivity, albeit with reduced potency (the structures of the molecules and their in vitro efficacy against CK2 are shown in Figure 1).

### 2.1. Inhibition of CK2 Substrates with CX-4945 and SGC-CK2-2 Reveals Target-Specific Sensitivities

We treated HeLa cells, a human cervical cancer line, with increasing doses of CX-4945 and SGC-CK2-2 for 24 h. To verify the efficacy of the inhibitors, we monitored the phosphorylation of two well-characterized CK2 substrates: S129Akt and S13-cdc37. These substrates were selected based on their different responses to kinase inhibition [23]. S129Akt was described in 2015 as a specific CK2 substrate [29], and its phosphorylation is highly sensitive to CK2 inhibition showing a strong early reduction. On the contrary, S13-cdc37, described in 2004 as a CK2-specific substrate [30], requires higher inhibitor concentrations or longer exposure times to achieve a significant impact on its phosphorylation. The phosphorylation of both substrates is reduced in CK2-knockout cells [31,32]. Moreover, to gain a more comprehensive view of CK2 substrate phosphorylation, we used a phosphoantibody developed against p-Ser/p-Thr containing a CK2 consensus sequence (pS/pTDXE motif). Most signals detected by this antibody are sensitive to CK2 inhibition [23] and are significantly reduced in CK2-knockout cells [31,32], supporting the antibody’s efficacy in providing an overview of phosphorylation across multiple CK2 substrates and assessing the general inhibition rate of CK2 signaling.

**Figure 2:**
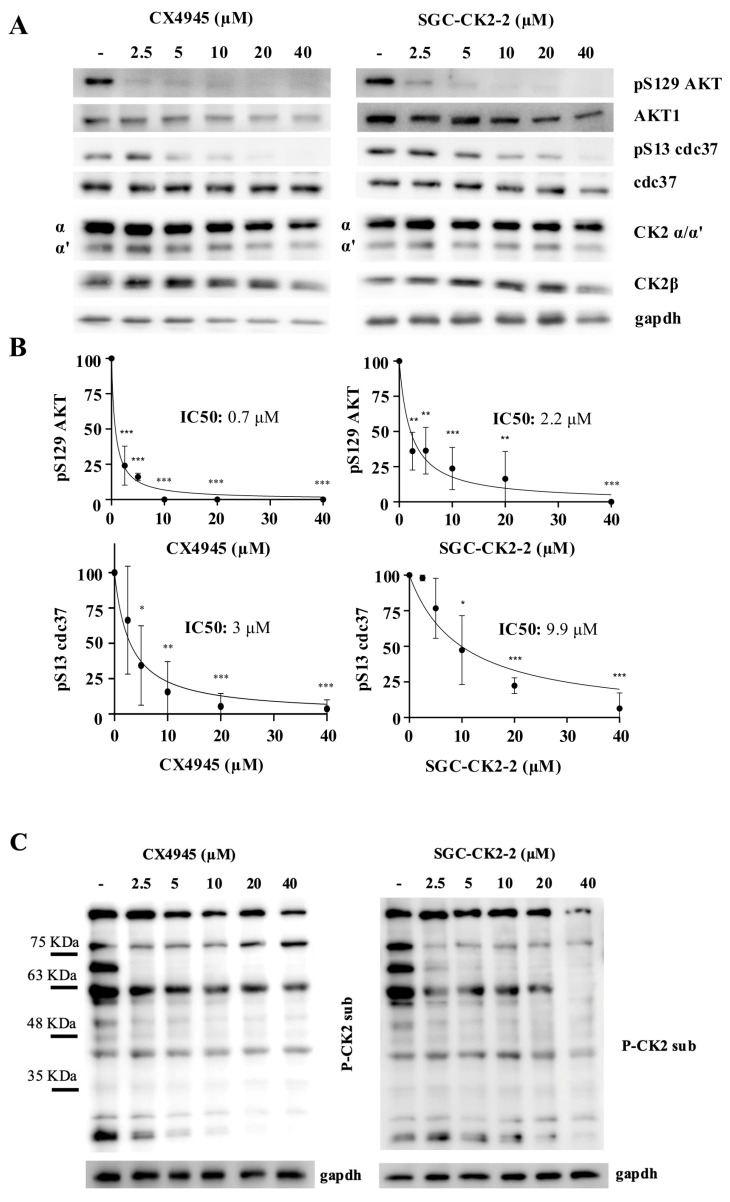
Dose effect of CX-4945 and SGC-CK2-2 on the phosphorylation of CK2 substrates in HeLa cells. (**A**) HeLa cells were treated for 24 h with vehicle (DMSO) (−) or increasing concentrations of CX-4945 or SGC-CK2-2 and then harvested. Protein lysates were analyzed by Western blot with the indicated antibodies. Anti-gapdh is the loading control. (**B**) Densitometric quantification of the immunostained bands of phospho-Akt1 S129 and phospho-cdc37 S13. Quantification of the bands relative to non-treated cells was set as 100 (means ± SD values, *n* = 3, Student’s *t-*test * *p* < 0.05; ** *p* < 0.01; *** *p* < 0.001). (**C**) HeLa cells were treated for 24 h with vehicle (DMSO) (−) or increasing concentrations of CX-4945 or SGC-CK2-2 and then harvested. Protein lysates were analyzed by Western blot with anti-phospho-CK2 substrates; gapdh was used as loading control.

In HeLa cells, we analyzed the dose-dependent effects of the two CK2 inhibitors. Phosphorylation of Akt at Ser129 was confirmed to be highly sensitive to CK2 inhibition, as it was completely abolished at the lowest concentration of CX-4945 tested (2.5 µM) (Figure 2A). Consistently, CX-4945 showed higher potency than SGC-CK2-2, with IC_50_ values of 0.7 and 2.2 µM, respectively (Figure 2B). In contrast, inhibition of Cdc37 phosphorylation required higher concentrations of both compounds (IC_50_ = 3 µM for CX-4945 and 9 µM for SGC-CK2-2) (Figure 2B). These results highlight that two different CK2 phospho-substrates may respond variably to kinase inhibition, with one being more responsive and the other requiring a higher concentration of the inhibitor. This conclusion was further supported by Western blot analysis using an antibody recognizing CK2 consensus p-Ser/p-Thr motifs (Figure 2B). The overall phosphorylation pattern revealed two main classes of CK2 substrates: one group exhibiting a marked decrease in phosphorylation at low inhibitor concentrations and another showing a gradual, dose-dependent reduction, with complete inhibition observed only at higher inhibitor levels.

**Figure 3:**
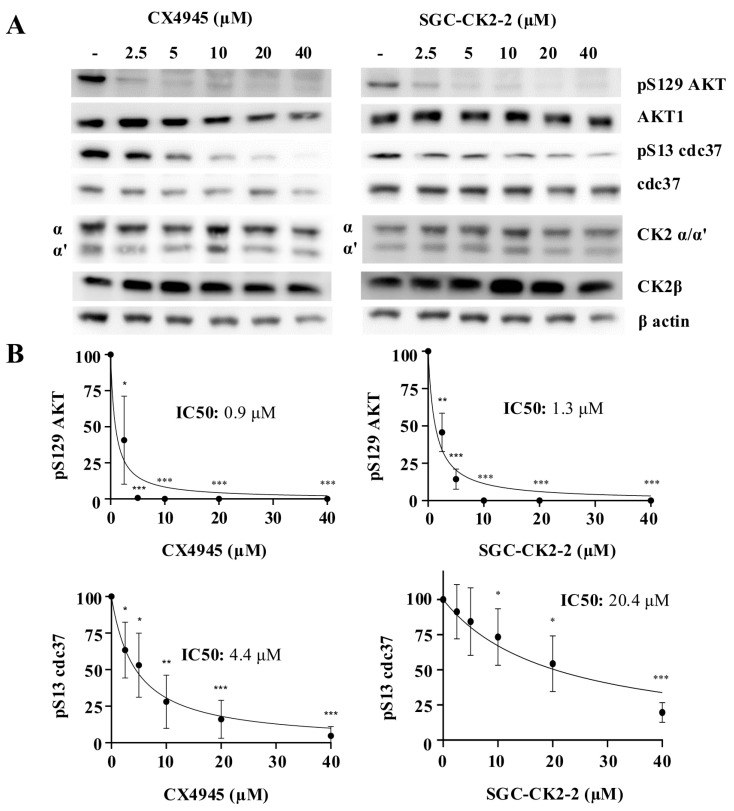

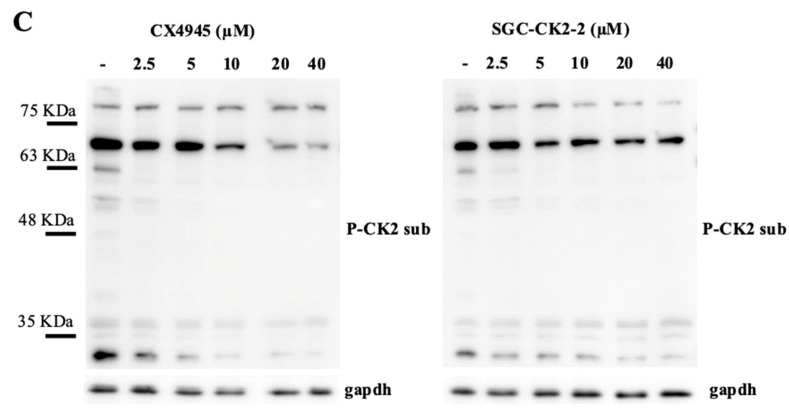
Dose effect of CX-4945 and SGC-CK2-2 on the phosphorylation of CK2 substrates in MDA-MB-231 cells. (**A**) MDA-MB-231 were treated for 24 h with vehicle (DMSO) (−) or increasing concentrations of CX-4945 or SGC-CK2-2 and then harvested. Protein lysates were analyzed by Western blot with the indicated antibodies. Anti-gapdh is the loading control. (**B**) Densitometric quantification of the immunostained bands of phosphor-Akt1 S129 and phospho-cdc37 S13. Quantification of the bands relative to non-treated cells was set as 100 (means ± SD values, *n* = 3, Student’s *t*-test * *p* < 0.05; ** *p* < 0.01; *** *p* < 0.001). (**C**) MDA-MB-231 cells were treated for 24 h with vehicle (DMSO) (−) or increasing concentrations of CX-4945 or SGC-CK2-2 and then harvested. Protein lysates were analyzed by Western blot with anti-phospho-CK2 substrates; gapdh was used as loading control.

We repeated the same treatments using a different cell line, breast cancer MDA-MB-231. Figure 3 shows how increased dosages of CX-4945 and SGC-CK2-2 affect CK2 phosphorylation in these cancer cells. Even though MDA-MB-231 was less responsive to the two CK2 inhibitors than HeLa cells, the overall outcome appeared similar. As stated, and confirmed by our results, CX-4945 was found again to be a more potent inhibitor than SGC-CK2-2. In detail, p-S129 Akt exhibited a suppression of the signal at lower concentrations of inhibitors: CX-4945 IC50 0.9 vs. SGC-CK2-2 IC50 1.3 μM. Conversely, p-S13 CDC-37 demonstrated responsiveness only at higher concentrations of inhibitors: CX-4945 IC50 4.4 vs. SGC-CK2-2 IC50 20.4 μM. A similar trend was observed using CK2 p-substrates antibody, with some p-sites exhibiting greater sensitivity to CK2 inhibition and others requiring higher concentrations of compounds.

### 2.2. CX-4945 Strongly Affects Cell Viability in a Dose-Dependent Manner Compared with SGC-CK2-2

Next, we evaluated HeLa and MDA-MB-231 cell viability using the same concentrations of inhibitors applied for suppress CK2 signaling.

**Figure 4:**
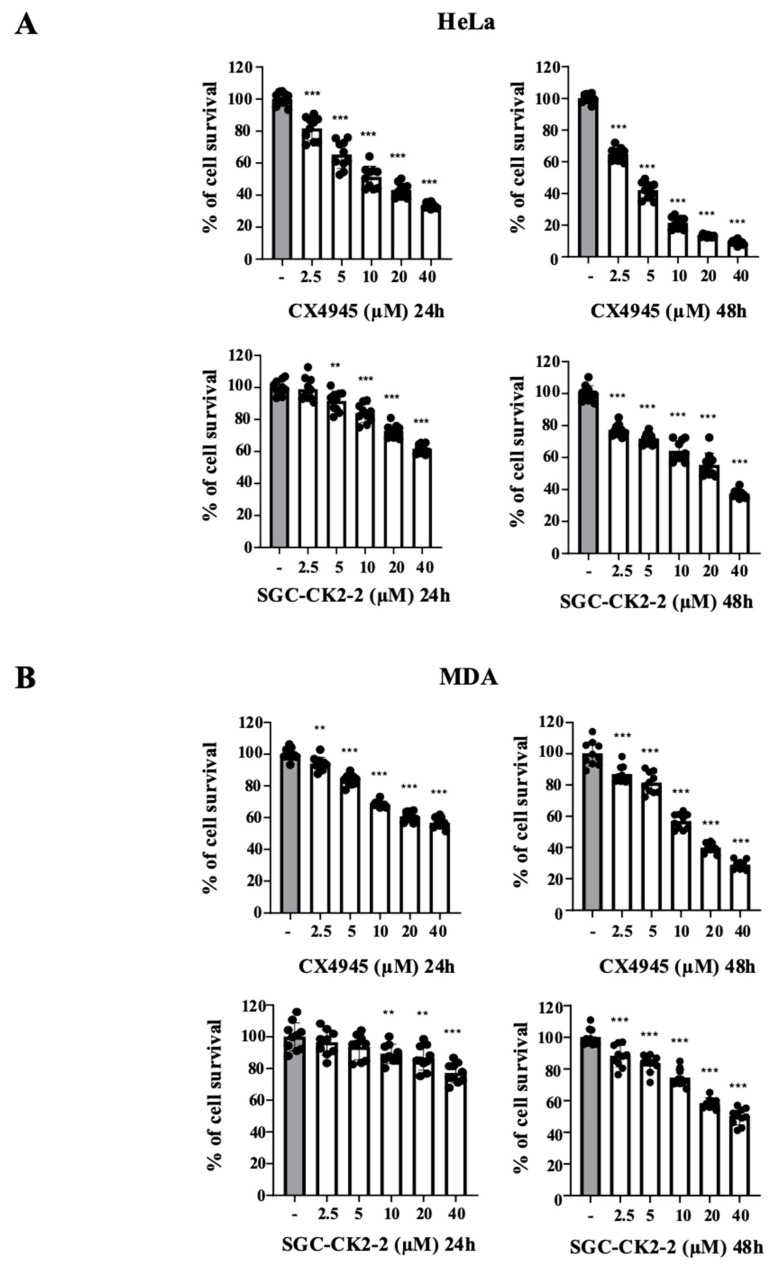
Dose effect of CX-4945 and SGC-CK2-2 on HeLa and MDA cell proliferation. The viability of HeLa (**A**) or MDA-MB-231 (**B**) cells was assessed by the MTT assay after 24 h or 48 h treatment with vehicle (DMSO) (−) or increasing concentrations of CX-4945 or SGC-CK2-2 and expressed as a percentage (means ± SD values, *n* ≥ 3; Student’s *t*-test ** *p* < 0.01; *** *p* < 0.001 vs. Ctrl).

MTT assays, shown in Figure 4, reveal that CX-4945 exhibit a dose-dependent reduction in cell viability, with a stronger effect observed after 48 h of treatment. HeLa cells are more sensitive to the compound than MDA-MB-231 cells. Notably, although a concentration of 2.5 µM is sufficient to markedly reduce the phosphorylation of a large part of the CK2 substrates, including complete inhibition of Akt S129 phosphorylation, the effect on cell viability remains modest (~20% after 24 h and ~30% after 48 h in HeLa cells; ~5% after 24 h and ~15% after 48 h in MDA cells).

In contrast, SGC-CK2-2 exhibits weaker cytotoxic activity than CX-4945. After 24 h of treatment, it does not achieve a 50% reduction in cell viability in either HeLa or MDA-MB-231 cells. Even at concentrations that nearly abolish Akt S129 phosphorylation (2.5 µM in HeLa and 5 µM in MDA, 24 h treatment), no significant effect on cell viability is detected. However, at higher doses, SGC-CK2-2 produces a dose-dependent inhibition of cell viability, with stronger cytotoxic responses evident after 48 h. The lower efficacy of SGC-CK2-2 compared with CX-4945 is consistent with its weaker inhibition of CK2 activity.

To further confirm our data on CK2 inhibitors’ impact on cell viability, we quantified the amount of the nuclear protein poly (ADP-ribose) polymerase 1 (PARP-1) by Western blotting. Indeed, PARP-1 (116 KDa) is cleaved during apoptosis, generating fragments of 89 kDa and 24 kDa. The quantification of the 89 kDa fragment is commonly used as an apoptotic marker.

**Figure 5:**
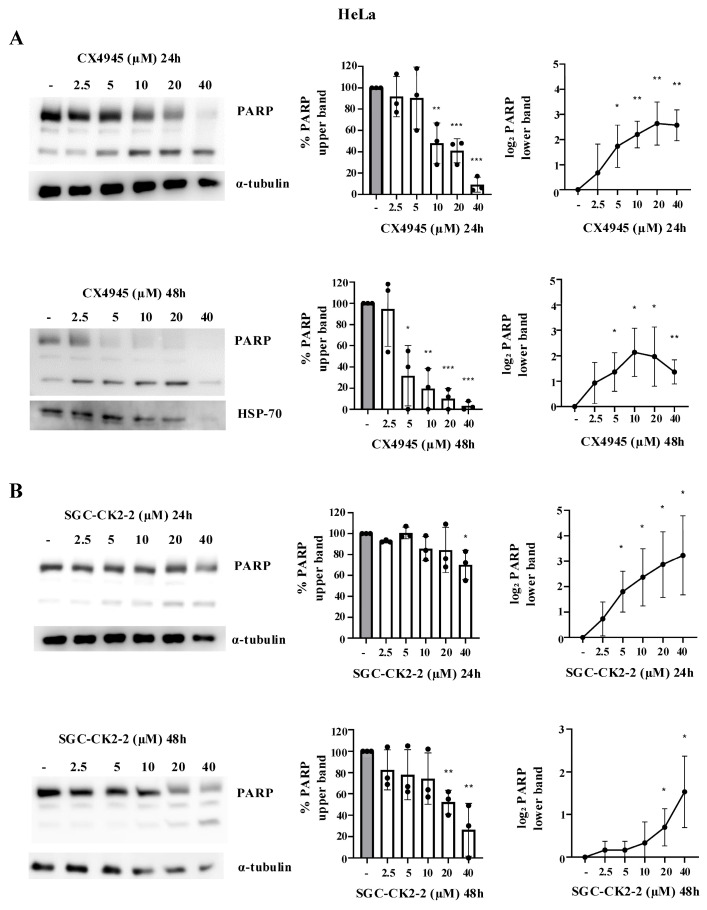
Dose effect of CX-4945 and SGC-CK2-2 on PARP-1 in HeLa cells. HeLa cells were treated with vehicle (DMSO) (−) or increasing concentrations of CX-4945 (**A**) or SGC-CK2-2 (**B**) for 24 h or 48 h and then harvested. Protein lysates were analyzed by Western blot with anti-PARP antibody. α-Tubulin and HSP-70 were used as loading control. On the right side of the figure, densitometric quantification of the immunostained bands (upper bands expressed in percentage and lower bands expressed in log_2_) of PARP (means ± SD values, *n* = 3; Student’s *t*-test * *p* < 0.05; ** *p* < 0.01; *** *p* < 0.001 vs. Ctrl).

In HeLa cells (Figure 5A) treated with CX-4945, PARP-1 degradation was visible at 10 µM after 24 h and at 5 µM after 48 h of treatment. Moreover, the 89 KDa fragment was already detected even at lower concentrations: 5 µM after 48 h and 2.5 µM after 48 h. Of note, the 2.5 µM concentration of CX-4945 previously led to an almost complete dephosphorylation of S129 Akt and of a large part of CK2 p-substrates. However, no significant detection of PARP cleavage was observed at 24 h of treatment, with the 89 KDa fragment visible at these concentrations only after 48 h of treatment.

In SGC-CK2-treated HeLa cells, a reduction in total PARP-1 levels was observed only at the highest concentration used (40 µM) after 24 h of treatment and for a lower dose (20 µM) after 48 h (Figure 5B). Again, the dephosphorylation of S129 Akt and of a large part of CK2 p-substrates was observed at a lower concentration of inhibitors (Figure 2).

**Figure 6:**
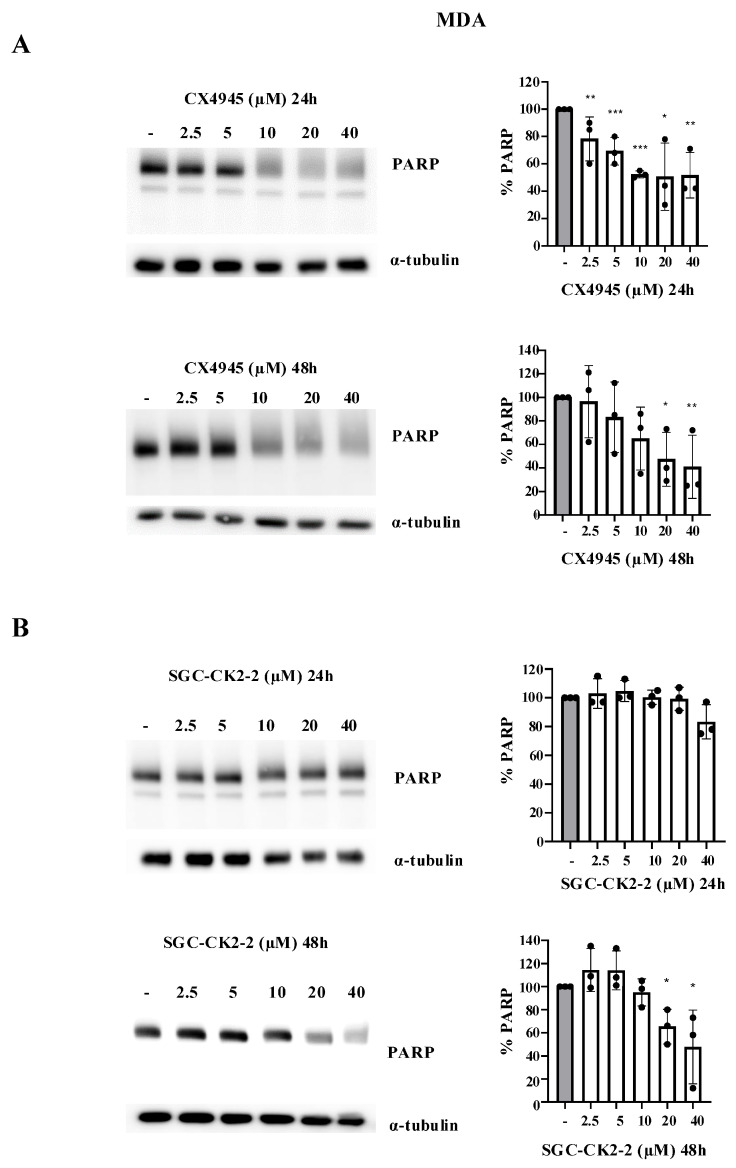
Dose effect of CX-4945 and SGC-CK2-2 on PARP-1 in MDA-MB-231 cells. MDA-MB-231 cells were treated with vehicle (DMSO) (−) or increasing concentrations of CX-4945 (**A**) or SGC-CK2-2 (**B**) for 24 h or 48 h and then harvested. Protein lysates were analyzed by Western blot with anti-PARP antibody. α-Tubulin and HSP-70 were used as loading control. On the right side of the figure, densitometric quantification of the immunostained bands (upper bands expressed in percentage and lower bands expressed in log_2_) of PARP (means ± SD values, *n* = 3; Student’s *t*-test * *p* < 0.05; ** *p* < 0.01; *** *p* < 0.001 vs. Ctrl).

Qualitatively, similar results were observed following the treatment of MDA cells. Notably, in MDA-MB-231 cells, the 89 kDa fragment was not detected following inhibitor treatment, whereas a decrease in total PARP-1 levels was clearly observed (Figure 6). As previously observed (Figure 4B), MDA-MB-231 cells appear to be more resistant to cell death induction with both inhibitors. In particular, at all concentrations tested, SGC-CK2-2 did not induce any degradation of PARP-1 at 24 h (concentrations sufficient to abolish the phosphorylation of most CK2 phospho-substrates), whereas significant degradation was observed only at 48 h and at concentrations of 20 µM or higher.

## 3. Discussion

In this paper we compare the effects of SGC-CK2-2 and its parental compound, CX-4945, on CK2 signaling and cell viability in MDA-MB-231 and HeLa cells. As previously mentioned, SGC-CK2-2 is a more specific compound than CX-4945, a feature that has been achieved with a partial loss in potency (Figure 1). Notably, SGC-CK2-2, although requiring higher concentrations than CX-4945, is still capable of completely suppressing CK2 signaling. A limitation of our study is that all experiments were performed in vitro, and therefore, the clinical translational value cannot be fully addressed at this stage. While detailed in vitro analyses such as those presented here are essential as a first step to define the compound’s specificity and cellular effects, future studies will need to evaluate the in vivo pharmacological properties of SGC-CK2-2 (in particular, pharmacokinetic data are not yet provided), to assess its impact on tumor growth either alone or in combination with other agents, and to further clarify the potential of CK2 as a therapeutic target in cancer.

Our dose–response analysis of these inhibitors on CK2 p-substrates highlights an important concept: the CK2 substratome displays varying sensitivity to different inhibitor concentrations. Several factors may contribute to this variability. Different substrates may have distinct affinities for CK2, with some being efficiently phosphorylated even when only low levels of active kinase remain. Again, the differential stability of phosphosites, due to varying turnover rates or sensitivity to phosphatases, can only be fully overcome by achieving different degrees of kinase inhibition. Additionally, the subcellular distribution of both the kinase and the inhibitor can differ. Depending on the physical and chemical properties of the inhibitor and its concentration, the inhibitory effect may not be uniform across all cellular compartments. As a result, kinase activity could be more effectively suppressed in some regions of the cell than in others, leading to differential effects on substrate phosphorylation. It is likely that the differential sensitivity observed is not explained by a single mechanism but rather by a combination of factors. Regardless of the underlying mechanisms, this finding underscores an important limitation: monitoring a single phosphosite is not always sufficient to accurately assess kinase inhibition. For example, in our study, the inhibitor concentration required to completely abolish phosphorylation of Akt at S129 was not enough to significantly reduce phosphorylation of other CK2 substrates or to affect cell viability.

This observation has crucial implications for evaluating kinase inhibitors. If we were to assess CK2 inhibition based solely on the minimal concentration needed to block S129 Akt phosphorylation and its effect on cell death, we might conclude that complete CK2 inhibition does not impact cell viability. However, a broader analysis of the entire CK2 substratome presents a different picture. Using higher concentrations of inhibitors able to abolish more-resilient CK2 phosphosites, we observe a dose-dependent response in cell death, evidenced by reduced cell proliferation (MTT assay) and enhanced apoptosis (PARP-1). These results are consistent with findings in experiments with CK2-deficient C2C12 cells, which display a proliferation rate similar to wild-type (WT) cells. Although these cells completely lack CK2α, they express an N-terminally truncated form of CK2α’ that accounts for less than 10% of total CK2 activity. Our previous studies have shown that this minimal CK2 activity is sufficient to sustain cell proliferation [33]. These and previous results [25,27] suggest that partial CK2 inhibition may have little impact on cell proliferation at least in some cell lines. Notably, the effect of CK2 inhibition on cell viability appears to be strongly cell type dependent [25].

Summarizing, our results emphasize the importance of comprehensive kinase signaling assessments. Specifically, when evaluating kinase involvement in a given pathway, it is essential to confirm the suppression of p-substrates directly implicated in that pathway rather than relying on a single phosphosite as a marker of inhibition.

## 4. Materials and Methods

### 4.1. Materials

Protease inhibitor cocktail was from Calbiochem (Darmstadt, Germany), while phosphatase inhibitor cocktails 2 and 3 were from Merck (Darmstadt, Germany). Anti-CK2α/CK2α’ (MCA3031Z) antibody was from Bio-Rad Laboratories (Hercules, CA, USA). Anti-CK2β (76025), anti-phospho-Akt1 S129 (133458), and anti-phospho-cdc37 S13 (EPR4879) antibodies were from Abcam (Cambridge, UK). Anti-β-actin antibody (A2228) and anti-α-tubulin (T5168) were purchased from Merck (Darmstadt, Germany). Anti-Akt1 (sc-5298), anti-cdc37 (E-4), anti-gapdh (0411), and anti-HSP-70 (3A3) antibodies were from Santa Cruz Biotechnology (Dallas, TX, USA). Anti-PARP was from Roche (Basel, Switzerland), while anti-phospho-CK2 substrates were from Cell Signaling (Danvers, MA, USA).

Secondary antibodies towards rabbit and mouse IgG, conjugated to horseradish peroxidase, were from PerkinElmer (Waltham, MA, USA).

### 4.2. Cell Culture

HeLa cells were provided by Prof. Harald Stenmark, University of Oslo, Norway. MDA-MB-231 cells were provided by Prof. Vincent S. Tagliabracci, University of Texas, USA. HeLa and MDA-MB-231 cells were maintained in 5% CO_2_ in DMEM (D6546, Merck, Darmstadt, Germany) supplemented with 10% FBS (FBS-HI-12A, Capricorn Scientific, Ebsdorfergrund, Germany), 2 mM L-glutamine, 100 U/mL penicillin, and 100 mM streptomycin (G1146, Merck, Darmstadt, Germany) in an atmosphere containing 5% CO_2_.

Cells were treated for 24 h or 48 h with vehicle (DMSO) (−) or increasing concentrations of CX-4945 (MedChemExpress-Monmouth Junction, NJ, USA) or SGC-CK2-2 (SML3909, Merck, Darmstadt, Germany) dissolved in dimethyl sulfoxide (DMSO, D2650, Merck (Darmstadt, Germany).

### 4.3. Cell Lysis and Western Blotting

Cells were detached with trypsin (59417C, Merck, Darmstadt, Germany), washed with PBS (omniPur PBS tablets 6501, EMD Millipore Corporation, Darmstadt, Germany), and lysed for 20 min on ice in the lysis buffer containing 20 mM Tris–HCl (pH 8.0), 150 mM NaCl, 2 mM EDTA, 2 mM EGTA, 1% Triton X-100 (*v*/*v*), protease inhibitor cocktail Complete (Roche), and phosphatase inhibitor cocktail 2 and 3 (Merck, Darmstadt, Germany). Cell lysates were centrifuged at 10,000× *g* for 10 min at 4 °C. The supernatant was collected, and protein concentration was determined by the Bradford method. Equal amounts of total protein extracts were loaded on SDS-PAGE, blotted on Immobilon-P membranes (Millipore), processed by Western blot with the indicated antibody, and detected by chemiluminescence on ImageQuant LAS 500 (GE Healthcare Life Sciences, Chicago, IL, USA).

### 4.4. MTT Assay

Cellular growth was evaluated by MTT (3-(4,5-dimethylthiazol-2-yl)-2,5-diphenyltetrazolium bromide) reduction assay, incubating 1 × 10^4^ cells in a 96-well plate with increasing doses of CX-4945 and SGC-CK2-2 (2, 5, 5, 10, 20, and 40 µM) for 24 h or 48 h, with at least three replicate wells for each condition. One hour before the end of the incubations, 10 μL of MTT solution (5 mg/mL in PBS) was added to each well. Incubations were blocked by the addition of 20 μL of a stop solution (20% SDS, 50% N,N-dimethylformamide, 2% acetic acid, and 25 mM HCl, pH 4.7). Plates were read at λ 540 nm absorbance, in a Titertek Multiskan Plus plate reader (Flow Laboratories, Sutton, UK).

### 4.5. Statistical Analysis

Results are presented as mean ± SD. The statistical significance was calculated using the *t*-test. Differences were considered statistically significant with *p* < 0.01 or *p* < 0.05. In instances in which the measured value was zero, an arbitrary minimal value of 1 was assigned in order to enable statistical analysis. This adjustment did not affect the interpretation of the results, as it was applied consistently across all datasets. All statistical analyses and graphs were produced using Prism (GraphPad 4.0 Software).

## Data Availability

The original contributions presented in this study are included in the article. Further inquiries can be directed to the corresponding authors.

## References

[1] Borgo C., D’Amore C., Sarno S., Salvi M., Ruzzene M. (2021). Protein Kinase CK2: A Potential Therapeutic Target for Diverse Human Diseases. Signal Transduct. Target. Ther..

[2] Strum S.W., Gyenis L., Litchfield D.W. (2022). CSNK2 in Cancer: Pathophysiology and Translational Applications. Br. J. Cancer.

[3] Trembley J.H., Kren B.T., Afzal M., Scaria G.A., Klein M.A., Ahmed K. (2023). Protein Kinase CK2—Diverse Roles in Cancer Cell Biology and Therapeutic Promise. Mol. Cell. Biochem..

[4] Pandit V., DeGeorge K., Nohe A. (2024). Scoping Pleiotropy of CK2 in Musculoskeletal Disorders for a Novel Targeting Approach. Kinases Phosphatases.

[5] Quezada Meza C.P., Ruzzene M. (2023). Protein Kinase CK2 and SARS-CoV-2: An Expected Interplay Story. Kinases Phosphatases.

[6] Baier A., Szyszka R. (2022). CK2 and Protein Kinases of the CK1 Superfamily as Targets for Neurodegenerative Disorders. Front. Mol. Biosci..

[7] Ampofo E., Nalbach L., Menger M.D., Montenarh M., Götz C. (2019). Protein Kinase CK2-A Putative Target for the Therapy of Diabetes Mellitus?. Int. J. Mol. Sci..

[8] Yang W., Wei H., Benavides G.A., Turbitt W.J., Buckley J.A., Ouyang X., Zhou L., Zhang J., Harrington L.E., Darley-Usmar V.M. (2022). Protein Kinase CK2 Controls CD8+ T Cell Effector and Memory Function during Infection. J. Immunol..

[9] Silva-Pavez E., Tapia J.C. (2020). Protein Kinase CK2 in Cancer Energetics. Front. Oncol..

[10] Buontempo F., McCubrey J.A., Orsini E., Ruzzene M., Cappellini A., Lonetti A., Evangelisti C., Chiarini F., Evangelisti C., Barata J.T. (2018). Therapeutic Targeting of CK2 in Acute and Chronic Leukemias. Leukemia.

[11] Villalobos-Nova K., Toro M.d.l.Á., Pérez-Moreno P., Niechi I., Tapia J.C. (2024). The CK2/ECE1c Partnership: An Unveiled Pathway to Aggressiveness in Cancer. Kinases Phosphatases.

[12] Chua M.M.J., Ortega C.E., Sheikh A., Lee M., Abdul-Rassoul H., Hartshorn K.L., Dominguez I. (2017). CK2 in Cancer: Cellular and Biochemical Mechanisms and Potential Therapeutic Target. Pharmaceuticals.

[13] Ong H.W., Drewry D.H., Axtman A.D. (2023). CK2 Chemical Probes: Past, Present, and Future. Kinases Phosphatases.

[14] Day-Riley S., West R.M., Brear P.D., Hyvönen M., Spring D.R. (2024). CK2 Inhibitors Targeting Inside and Outside the Catalytic Box. Kinases Phosphatases.

[15] Siddiqui-Jain A., Drygin D., Streiner N., Chua P., Pierre F., O’Brien S.E., Bliesath J., Omori M., Huser N., Ho C. (2010). CX-4945, an Orally Bioavailable Selective Inhibitor of Protein Kinase CK2, Inhibits Prosurvival and Angiogenic Signaling and Exhibits Antitumor Efficacy. Cancer Res..

[16] (2017). CX-4945 Granted Orphan Drug Designation. Oncol. Times.

[17] Son Y.H., Song J.S., Kim S.H., Kim J. (2013). Pharmacokinetic Characterization of CK2 Inhibitor CX-4945. Arch. Pharmacal Res..

[18] D’Amore C., Borgo C., Sarno S., Salvi M. (2020). Role of CK2 Inhibitor CX-4945 in Anti-Cancer Combination Therapy—Potential Clinical Relevance. Cell. Oncol..

[19] Grygier P., Pustelny K., Nowak J., Golik P., Popowicz G.M., Plettenburg O., Dubin G., Menezes F., Czarna A. (2023). Silmitasertib (CX-4945), a Clinically Used CK2-Kinase Inhibitor with Additional Effects on GSK3β and DYRK1A Kinases: A Structural Perspective. J. Med. Chem..

[20] Menyhart D., Gyenis L., Jurcic K., Roffey S.E., Puri A., Jovanovic P., Szkop K.J., Pittock P., Lajoie G., Axtman A.D. (2023). Comparison of CX-4945 and SGC-CK2-1 as Inhibitors of CSNK2 Using Quantitative Phosphoproteomics: Triple SILAC in Combination with Inhibitor-Resistant CSNK2. Curr. Res. Chem. Biol..

[21] Kim H., Choi K., Kang H., Lee S.-Y., Chi S.-W., Lee M.-S., Song J., Im D., Choi Y., Cho S. (2014). Identification of a Novel Function of CX-4945 as a Splicing Regulator. PLoS ONE.

[22] Agnew C., Liu L., Liu S., Xu W., You L., Yeung W., Kannan N., Jablons D., Jura N. (2019). The Crystal Structure of the Protein Kinase HIPK2 Reveals a Unique Architecture of Its CMGC-Insert Region. J. Biol. Chem..

[23] Cesaro L., Zuliani A.M., Bosello Travain V., Salvi M. (2023). Exploring Protein Kinase CK2 Substrate Recognition and the Dynamic Response of Substrate Phosphorylation to Kinase Modulation. Kinases Phosphatases.

[24] Marin O., Meggio F., Draetta G., Pinna L.A. (1992). The Consensus Sequences for Cdc2 Kinase and for Casein Kinase-2 Are Mutually Incompatible. A Study with Peptides Derived from the Beta-Subunit of Casein Kinase-2. FEBS Lett..

[25] Wells C.I., Drewry D.H., Pickett J.E., Tjaden A., Krämer A., Müller S., Gyenis L., Menyhart D., Litchfield D.W., Knapp S. (2021). Development of a Potent and Selective Chemical Probe for the Pleiotropic Kinase CK2. Cell Chem. Biol..

[26] Yang X., Ong H.W., Dickmander R.J., Smith J.L., Brown J.W., Tao W., Chang E., Moorman N.J., Axtman A.D., Willson T.M. (2023). Optimization of 3-Cyano-7-Cyclopropylamino-Pyrazolo[1,5-a]Pyrimidines toward the Development of an In Vivo Chemical Probe for CSNK2A. ACS Omega.

[27] Davis-Gilbert Z.W., Krämer A., Dunford J.E., Howell S., Senbabaoglu F., Wells C.I., Bashore F.M., Havener T.M., Smith J.L., Hossain M.A. (2023). Discovery of a Potent and Selective Naphthyridine-Based Chemical Probe for Casein Kinase 2. ACS Med. Chem. Lett..

[28] Salvi M., Borgo C., Pinna L.A., Ruzzene M. (2021). Targeting CK2 in Cancer: A Valuable Strategy or a Waste of Time?. Cell Death Discov..

[29] Di Maira G., Salvi M., Arrigoni G., Marin O., Sarno S., Brustolon F., Pinna L.A., Ruzzene M. (2005). Protein Kinase CK2 Phosphorylates and Upregulates Akt/PKB. Cell Death Differ..

[30] Miyata Y., Nishida E. (2004). CK2 Controls Multiple Protein Kinases by Phosphorylating a Kinase-Targeting Molecular Chaperone, Cdc37. Mol. Cell. Biol..

[31] Franchin C., Borgo C., Cesaro L., Zaramella S., Vilardell J., Salvi M., Arrigoni G., Pinna L.A. (2018). Re-Evaluation of Protein Kinase CK2 Pleiotropy: New Insights Provided by a Phosphoproteomics Analysis of CK2 Knockout Cells. Cell. Mol. Life Sci..

[32] Lettieri A., Borgo C., Zanieri L., D’Amore C., Oleari R., Paganoni A., Pinna L.A., Cariboni A., Salvi M. (2019). Protein Kinase CK2 Subunits Differentially Perturb the Adhesion and Migration of GN11 Cells: A Model of Immature Migrating Neurons. Int. J. Mol. Sci..

[33] Borgo C., D’Amore C., Cesaro L., Itami K., Hirota T., Salvi M., Pinna L.A. (2020). A N-Terminally Deleted Form of the CK2α’ Catalytic Subunit Is Sufficient to Support Cell Viability. Biochem. Biophys. Res. Commun..

